# The elusive ideal of inclusiveness: lessons from a worldwide survey of neurologists on the ethical issues raised by whole-genome sequencing

**DOI:** 10.1186/s12910-017-0187-8

**Published:** 2017-04-11

**Authors:** Thierry Hurlimann, Iris Jaitovich Groisman, Béatrice Godard

**Affiliations:** Institut de recherche en santé publique (IRSPUM), Omics-Ethics Research Group, University of Montreal, PO Box 6128, Station Centre-ville, Montreal, QC H3C 3 J7 Canada

**Keywords:** Research ethics, Justice, Fair inclusion, Genomics

## Abstract

The anticipation of ethical issues that may arise with the clinical use of genomic technologies is crucial to envision their future implementation in a manner sensitive to local contexts. Yet, populations in low- and middle-income countries are underrepresented in studies that aim to explore stakeholders’ perspectives on the use of such technologies. Within the framework of a research project entitled “Personalized medicine in the treatment of epilepsy”, we sought to increase inclusiveness by widening the reach of our survey, inviting neurologists from around the world to share their views and practices regarding the use of whole-genome sequencing in clinical neurology and its associated ethics. We discuss herein the compelling scientific and ethical reasons that led us to attempt to recruit neurologists worldwide, despite the lack, in many low- or middle-income countries, of access to genomic technologies. Recruitment procedures and their results are presented and discussed, as well as the barriers we faced. We conclude that inclusive recruitment remains a challenging, albeit necessary and legitimate, endeavour.

## Background

Genome Canada and Génome Québec granted support to a research project entitled *“Personalized medicine in the treatment of epilepsy”* [1]. While the search for epilepsy genes has allowed the identification of several genes in idiopathic generalized epilepsy, as well as in syndromic epilepsies, determining genetic contributions to common epilepsies is challenging and much remains to be learned [2–5]. Next-generation sequencing technologies (NGS), such as whole-genome sequencing (WGS) and whole-exome sequencing (WES), offer a powerful tool in research on the genetics/genomics of epilepsy [5, 6]. Some phases of the project aimed at assessing the clinical utility of NGS as a diagnostic tool, in particular for pharmaco-resistant epilepsies. The project also sought to assess the societal and ethical issues that arise at the juncture of genomics research and personalized treatments. It notably examined neurologists’ views on the use of WGS in their practice, as well as the practical and ethical issues that may surround WGS and the return of its results to patients.

An internet-based survey was conducted to document neurologists’ views and practices on six main topics:

- Use of genetic testing (including use of WGS) in clinical practice;
- Circumstances in which WGS should be offered to patients;
- Concerns about the use of WGS in clinical practice;
- Potential benefits of the use of WGS in clinical practice;
- Return of WGS results;
- Needs concerning training and/or resources in genomics/genetics.

The survey was developed in collaboration with the neurologists involved in the clinical part of the research project. It was shortened and slightly modified after being pre-tested by six other neurologists in Canada. The estimated time to fill out the questionnaires was 15 min.

We invited neurologists from low-, middle-, and high-income countries to participate in this survey. This paper comments on the reasons that led us to attempt to reach for such inclusiveness, the challenges we faced, and the lessons we learned.

## Why include neurologists from low- to middle-income countries in such a project?

For a long time, the principles of justice and equity in research ethics have centered on the protection of marginalized and vulnerable populations (such as children, minorities, and pregnant women), yet with a pernicious effect: the protection afforded to vulnerable people has significantly prevented the latter from gaining a fair access to clinical studies [7]. This issue has been debated, particularly in the field of *clinical* research, where a lack of representation can affect the external validity of results, which in turn may increase health disparities between populations or communities [8]. Fair selection of participants in any kind of research project, *clinical or not*, is an important ethical requirement. It has become increasingly debated, notably in the field of global health research, which aims to promote greater equity worldwide. As stressed by the Canadian Coalition for Global Health Research, equity cannot be achieved without proactively and intentionally providing opportunities for “other voices” and diverse people to participate in research processes [9].

Ultimately, in any research project, research ethics boards have to ensure that there is a valid, reasonable, and scientifically grounded reason which justifies the exclusion of individuals from the opportunity to participate in research protocols. Yet, to what extent is such an ethical requirement achievable, or, in certain circumstances, even desirable?

The decision to include neurologists from around the world in our survey resulted from a process with cascading effects, which started with the suggestion, made by our funding agency, to include European neurologists in our survey. The World Health Organization (WHO) regional office for Europe counts 53 countries/geographical areas [10], among which, several are lower-middle to upper-middle-income areas, according to the World Bank classification [11]. In order to achieve fair inclusiveness in the selection of our participants, the question was raised whether such countries should be included or not, and if so, whether (and why) we could ignore countries in other regions of the world. While we expected that recruiting neurologists in many low- to middle-income countries might be challenging, as neurologic expertise [12, 13] and access to genomic technologies are limited or non-existent in many of the poorest countries, we had no legitimate reason not to try. On the contrary, many factors induced us to include all countries, such as explained below.

The economic and social impact of various neurological disorders such as stroke, Alzheimer’s and Parkinson’s diseases, and epilepsy in developing countries is high [14, 15]. Nearly 80% of the people with epilepsy live in low- to middle-income countries, and three fourths of them may not receive the treatment they need (treatment gap) [16]. Research and innovation gaps between emerging and developed countries are still wide, and most of the developing countries must rely on other more developed countries for funding and developing capacities [13]. The factors that may explain a slow recognition and implementation of improved care for people with neurological disorders in low- to middle-income countries are various, including resources-limited settings, unavailability of medications, lack of epidemiological data, the perception that neurologic disorders are too complex to address, a failure to recognize the cost of inaction, the stigma associated with the disorders, and cultural beliefs about their causation [17–19].

Researchers and international organizations have thus been calling for greater recognition from health agencies and more collaborative research to address the management of epilepsy in the developing world [13, 18]. According to WHO, projects that aim to reduce the treatment gap and morbidity of people with epilepsy, train health professionals, and develop models integrating epilepsy control into local health systems are ongoing in many countries [16]. This also includes projects involving the use of technology that may not be accessible in the poorest countries, such as is the case for the development of a mobile application to help “non-doctors” to diagnose (or prevent them from misdiagnosis of) epileptic seizures [20].

At the same time, the involvement of low- to middle-income countries in genomic research has been much debated, with explicit calls for the development of strategies at the local, national, regional and global levels, to encourage the production, dissemination, and use of genomic knowledge more equally, as well as to mitigate a genomic divide between developed and developing countries [21]. Debates have focused on clinical research, addressing, in particular, ethical challenges, such as priority setting, capacity building, community engagement, informed consent, ethical review, management of samples, and benefit-sharing (e.g., [22–25]) There are obvious ethical benefits to conducting genomic research on diseases affecting people in low- to middle-income countries, so as to reduce global health inequalities [22, 26]. This is particularly true in genetic research, where the findings resulting from studies conducted on specific populations, such as Caucasians, may not be relevant for other populations [2]. Although one may think that access to genetic technologies may not be affordable to the majority of populations in most low- to middle-income countries without international collaboration, progress has been made in this regard and ongoing genomic projects are conducted in a number of these countries [26–28].

Finally, several authors have stressed the importance of genetics in neurological disorders in the developing world (e.g., [29, 30]). An example of active research is the H3Africa project that focuses on the study of the complex interplay between environmental and genetic factors, which determines disease susceptibility (including hereditary neurological disorders) and drug responses in African populations [31]. In this context, the inclusion of neurologists located in low- to middle-income countries in a study aimed at assessing their views on the use of genomic technologies in their practice makes sense, independent of their actual access to these technologies. Anticipation of the ethical issues that could arise from the use of genomic technologies in low- to middle-income countries is crucial to envision their future use, develop sound partnerships, capacity building, and benefit-sharing which are sensitive to the local context and acceptance. For instance, our survey covers ethical issues, such as the need for resources, or still greater concerns about increased risks of stigma resulting from individuals’ genotyping. It is clearly worth exploring the perceptions of genetic testing in countries where neurological diseases are often stigmatizing conditions, when they are explained in terms of traditional beliefs [18, 32]. In such a context, presuming that neurologists in low- to middle-income countries would have little to say about the use of genomics technologies, notably because of a lack of access or expertise, is unacceptably paternalistic and biased.

## Recruiting neurologists worldwide: practical challenges

Our survey focused on the *clinical* use of WGS, so we could not limit ourselves to researchers. While email addresses of researchers can often be found on the internet through their publications or the website of the organizations they are affiliated to, public access to email addresses of physicians, whatever their specialty is seriously limited. One solution to also reach practitioners whose email addresses were not publicly accessible was to contact medical associations, colleges of physicians, and neurology associations/societies worldwide asking them to help us disseminate our invitation (which contained a hyperlink to the web-based survey) among their neurologist members. Procedures are further described in Table 1 legend. We identified 238 medical associations/colleges of physicians in 215 countries/geographical areas. Personalized emails were sent to 214 medical associations. These emails included the name of the contacted organization, its country or geographical region, a description of our study with a hyperlink to the survey, an estimate of the time needed to fill out the questionnaire, and a request to forward a letter of invitation to any neurologist members. Our emails stressed that access to, and expertise in, genetic technologies were not required to participate. A vast majority (91%) did not respond to our initial emails. Also, the initial reminders did not improve our response rate. Therefore, this recruitment procedure was abandoned before all reminders were sent. Thereafter, we focused exclusively on neurology associations.

**Table 1:** Contacts with medical associations, colleges of physicians and neurology associations

MEDICAL ASSOCIATIONS AND COLLEGES OF PHYSICIANS								
	# countries/areas listed^a^	# of organizations identified^b^	# of countries/areas for which NO relevant organization(s) could be identified	Contact and follow-up ^c^				
				YES^d^	NO^e^ or Lost in follow-up^f^	No answer	No contact email or invalid email addresses^c^	# of direct invitations to neurologists^g^
North America	3	30	0	1	7	22	0	-
Central America	6	8	1	0	0	8	0	-
South America	10	16	0	0	1	12	3	-
Caribbean	27	17	11	0	1	16	0	-
Europe (except Spain)^h^	53	65	3	0	7	54	4	-
Eastern Mediterranean	20	28	1	0	0	23	5	-
South-East Asia^h^	11	10	2	0	0	10	0	-
Western Pacific Region^h^	37	21	21	0	0	21	0	-
Africa	47	43	8	2	0	29	12	-
TOTAL (medical associations and colleges)	215	238	47	3	16	195	24	-
NEUROLOGY SOCIETIES AND ASSOCIATIONS								
International	-	3	-	2	0	1	0	0
North America	3	14	0	4	4	6	0	52
Central America	6	9	0	2	0	7	0	20
South America	10	17	0	8	2	7	0	0
Caribbean	27	2	25	2	0	0	0	97
Europe	54	87	7	29	3	54	1	323
Eastern Mediterranean	20	15	5	2	1	12	0	190
South-East Asia	11	14	4	0	1	11	2	110
Western Pacific region	37	15	25	3	0	12		38
Africa	47	14	35	1	0	12	1	0
TOTAL (neurology organizations)	215	190	101	53	11	122	4	830

^a^List of countries/geographical areas based on the WHO regions (http://www.who.int/countries/en/). Accessed Summer 2014

^b^Medical associations were identified through the Geneva Foundation for Medical Education and Research database: http://www.gfmer.ch/Medical_search/Medical_schools.php (Accessed Summer 2014). Search was completed with Google ([NAME OF THE COUNTRY] AND “medical association”] OR [NAME OF THE COUNTRY] AND “physician” and “college”. Neurology associations were identified through the World Federation of Neurology database at http://www.wfneurology.org/member-societies (Accessed Summer 2014) and the list of all the members of the European Academy of Neurology provided at https://www.ean.org/National-Neurological-Societies.2672.0.html (Accessed Summer 2014). Search was completed on Google with: [NAME OF THE COUNTRY] AND “neurology” (in Spanish, Portuguese, French or English). Neurosciences/neurosurgery organizations were excluded. Numbers include regional associations

^c^We sent personalized emails and one reminder using email address(es) provided on the websites mentioned in footnote (b) above. For medical associations: the sending of requests and reminders was interrupted during the process, given the very low response rate. For neurology organizations, in case of no answer or invalid email addresses, we consulted the neurology associations’ websites, Google and PubMed to identify names and email addresses of board members (President, Vice-president and Secretary). New personalized emails were sent instead of a reminder

^d^ Includes a few neurology associations that provided the complete list of their members’ emails

^e^Includes associations that refused to disseminate our invitation but forwarded our request or referred us to another association

^f^Organizations that gave a positive answer but 1) did not seem to proceed and could not be reached again; 2) required payment to disseminate our invitation and/or 3) would only communicate postal addresses of members

^g^ When the email addresses of neurology associations boards members (other than President/Vice-President/Secretary) were provided on websites, we sent a direct survey invitation to these members

^h^ Two medical associations were identified in Spain, but they were not contacted as the very low response rate had us interrupt this means of recruitment. Requests and reminders were likewise stopped for medical associations in South-East Asia and Western Pacific Region

We identified 190 neurology associations in 215 countries or geographical areas (Table 1). Email addresses could not be found or were invalid for four of them. Personalized emails and reminders were sent from September to December 2014. We attempted to contact the heads of these associations directly whenever possible, rather than using generic association accounts (see legend of Table 1, letter c). Sixty-five percent of these associations never answered, despite reminders and/or personalized emails to their board members. The 53 associations that agreed to help us were mainly located in Europe and South American countries. Despite the dissemination of our invitation by 53 associations, participation rates remained low (see Table 2). We subsequently sent personalized direct invitations and reminders to 830 neurologists whose email addresses were accessible on neurology association websites (such as board members or other contacts provided), as well as to 581 corresponding authors in clinical neurological research and 260 epileptologists, who were identified on the website of the International League Against Epilepsy (ILAE) (see Table 3). The survey was online for approximately nine months and was closed in May 2015. Our final sample was composed of 204 neurologists located mainly in Europe, South America, Central America, and the Caribbean (see Tables 3 and 4).

**Table 2:** Participation from October 2014 to April 2015

Date	Number of individuals accessing the web-based study
October 21, 2014	83
December 15, 2014	211^a^
March 2, 2015	233^a^
March 24, 2015	250^a^
April 7, 2015	257^a^
April 21, 2015	259^a^
May 2015	Final sample: 204^a^

^a^This number is higher than the final sample (n = 204), as we excluded respondents who exited the questionnaire after answering the first or the two first questions only

**Table 3:** Recruitment through publications and ILAE website

	Procedures	Total of invitations sent
Corresponding authors in clinical neurological research	• Pubmed search using ((neurolog*[Title]) AND clinic*[Title/Abstract]) AND patients[Title/Abstract], for the years 2012–2014, humans (excluding animals) • 697 publications identified • Identification of corresponding authors and email addresses • Personalized invitations + 2 reminders sent from October 2014 to February 11 2015	581 (all continents represented)
Epileptologists registered on ILAE website	• Identification of ILAE members • Personalized invitations + 2 reminders sent from February 9th to March 19 2015	260 (all continents represented)
		TOTAL: 841

**Table 4:** Respondents’ location

Respondents based in…	Number of respondents in countries^a^ (n)	Total *n* ^b^
Europe	Sweden (16); France (13); Spain (6); Portugal (6); UK (6); Lithuania (4); Croatia (2); *Georgia* (2); Italy (2); Norway (2); *Bulgaria* (1); Czech Republic (1); Estonia (1); Luxembourg (1); *Macedonia FYR* (1); *Kosovo* (1); *Romania* (1); *Serbia* (1); Switzerland (1); *Turkey* (1); *Ukraine* (1)	70
Central and South America + Caribbean	*Brazil* (21); Chile (9); *Peru* (9); Argentina (8); *Costa Rica* (2); *Colombia* (1); *Cuba* (1); *Dominican Republic* (1); *Guatemala* (1)	53
North America	Canada (11); *Mexico* (3); USA (1)	15
South-East Asia and Western Pacific Region	New Zealand (5); *Bangladesh* (2); China (2 in Hong Kong); Australia (1); *India* (1)	11
Eastern Mediterranean and Africa	*Lebanon* (2); *Tunisia* (1); *Rwanda* (1); *South Africa* (1)	5

^a^Italic: low-lower-middle- or upper-middle-income countries according to the World Bank: https://datahelpdesk.worldbank.org/knowledgebase/articles/906519 (Accessed August 26, 2016)

^b^50 respondents out of 204 did not answer this question

## Discussion

Response rates to Web surveys vary widely and depend on a variety of factors (e.g., characteristics of the audience, purpose of the survey, perceived benefit from participating in survey, incentives, length, convenience) [33]. Achieving high response rates when surveying physicians has always been a challenge [34, 35], as shown by the examples below. Helman and colleagues sought to determine why NGS was not broadly used by pediatric neurologists by surveying members of the Child Neurology Society during the 2015 Annual Meeting of the Society [3]. While the participation in the meeting was evaluated to be 1000 members [36], only 67 of them completed the survey (i.e., 6.5%). In 2012, Machini and colleagues sought to survey health care professionals who are likely to be involved in the implementation of WES and WGS [37]. Their attempts to recruit other health professionals than genetic counsellors proved unsuccessful. Middleton and colleagues achieved better participation rate in a survey aimed to gather the views of various stakeholders (worldwide) towards sharing incidental findings from whole-genome studies [38, 39]. However, they had to deploy an impressive combination of measures to disseminate their survey, such as advertisement on television, newspapers, internet (e.g., on Google), development of a website, hiring an independent film-maker to produce movies that provide the contextual information needed to answer the questions, creation of accounts in various social media (Facebook, Twitter, etc.), distribution of flyers, participation to congresses, direct invitations. They also conducted five pilot studies to develop their online survey [38]. Despite these efforts, their final sample of respondents was mainly composed of laypersons (i.e., public; 71%). Genetic/genomic professional researchers accounted for 17% of the sample, and “other health professional” - which include many others than physicians [40] - for 12%. We thus knew that we would face challenges in recruitment, given our limited resources.

Furthermore, “[c]ommitment to inclusion invites those involved in global health research to promote equity by proactively and intentionally providing opportunities for diverse people to be engaged in research processes” [9], [p. 5], as to avoid differentials in power among actors in the process. However, regarding our study, such engagement of neurologists or their association was not practically and financially feasible at a worldwide level. We acknowledge that a questionnaire may be shaped by researchers’ perceptions and values. It may contain concepts and categories that were influenced by such values, and as such may constitute the exercise of productive power [41, 42]. Thus, in order to give a voice to all neurologists worldwide, spaces for comments were added into the questionnaire, so as to avoid overly restrictive options for answers that would not fit into the reality of local contexts. Moreover, our survey was developed after extensive literature review of ethical issues that can arise in any location. Our letter of invitation as well as our emails to neurology associations emphasized that access to genetic technologies or expertise in this field was not needed to complete the survey. As we were not dealing with a vulnerable population, and given the nature of our project, we anticipated no risk for participants. In the opposite, we viewed this project as a beneficial step to produce results that could serve the aims of health equity if inclusion was broadened worldwide.

Ultimately, 53 neurology associations from around the world agreed to disseminate our invitation among their members, which suggests that the quality, or the local relevance of our questions, were not determinant factors for the high rate of non-response from the contacted associations. In addition, non-response was frequent in all countries, and from low- to high-income areas; thus we cannot simply infer that it was due to local specificities linked to the lack of expertise or access to genetic technologies. A vast majority of reactions from organizations that answered our first email or reminder, in particular in middle-income countries, were highly positive, although this enthusiasm did not translate into higher rates of local participation of neurologists. In all, 56 respondents (36%) were located in low- to middle-income countries (Table 4), demonstrating that neurologists in such countries actually saw an interest in participating. Conversely, participation rates were particularly low in North America, compared to Europe and Latin America. There are of course limitations to the interpretation of such results. Actual participation rates could not be calculated. We could not get information about the number of members of every participating association and had no control over the means used by participating organizations to disseminate our invitation (mass email to members, invitation published in a newsletter or on a website). Participation rates could clearly be impacted by these means of dissemination, as well as by snowball sampling procedures and the fact that some countries were more (or less) represented in the 1600 invitations we directly sent to neurologists in an attempt to improve the overall participation rate (Table 3). Furthermore, 50 respondents did not indicate their location.

In an analytical framework developed in 2008, Sarre and colleagues acknowledged the numerous challenges faced by researchers in the recruitment of organizations, practitioners and patients in primary care research [43]. While our study was not a clinical one, it required similar efforts to convince numerous medical and neurology organizations to get involved in our recruitment process. We were not expecting such a high rate of non-answer or refusal to cooperate from these associations. Most associations that explicitly refused to disseminate our invitation among their members invoked internal policies. In several cases, the associations explained that they could not contact their members for a research project in the development of which they had not been initially involved or in which they were not formally collaborating. Some associations would only provide us with postal addresses of their members and/or required payment. While rare, and only in high-income countries, in some cases we were notified that our emails or reminders were annoying, if not harassing. There are legitimate reasons for associations to act as gatekeepers, in order to avoid over or undue solicitation. This however constitutes a major barrier to participation. It means that many neurologists could not participate because they were not informed about our survey. To what extent this may constitute the exercise of structural power that may impede broader participation in research remains to be documented, as at the same time, our survey participation rate barely increased after sending more than 1600 direct invitations to neurologists worldwide (Table 3).

## Conclusion

We virtually screened the whole world, gathering web data to document the prevailing situation in the field of neurology in numerous countries, to help us interpret our results. It was crucial to give a voice to neurologists in countries that are most often excluded from such surveys, given economic and socio-demographic factors, as well as considerations about access to, and expertise in, genomic technologies. Such factors, notably in countries where genomic research is ongoing, obviously do not prevent neurologists from having valuable perspectives on the matters we were addressing in the survey. Ethical issues, such as local acceptability of genomic technologies and sound benefit-sharing in genomic research, must be anticipated, and this cannot be achieved without exploring the views of those who will be at the forefront of implementing and using such technologies.

As with most studies that involve the recruitment of health professionals, ideal inclusion has been a challenging endeavour. A possibility for greater success could have been to focus on one or two countries, and one or two neurology associations only. Yet, the low response rate in North America challenges such a viewpoint. From a global health research perspective, engagement of neurology associations in the development of the questionnaire itself could facilitate access to members, and overcome gate-keeping barriers.

It is important to acknowledge that researchers may face huge challenges in their efforts to achieve broad and fair inclusiveness, in any kind of research. This is also a lesson for those serving on research ethics boards: the ethical requirement of fair inclusion in research protocols must be balanced with practical difficulties, priority-setting in a context of limited funding resources, as well as with the potential risks that such a requirement can raise for the validity of results.

In global health research, “honouring th[e] principle [of inclusion] involves actively exploring ways to create opportunities for other voices, particularly for stakeholders who might not be immediately identified” [9], [p. 5]. Did we aim too high by attempting a worldwide recruitment? We think that in a global research effort, responsibilities must be distributed among actors and all have to contribute, as illustrated by this Amerindian legend:

*One day, a huge fire started in a forest that was home to several animal species. All of them were terrified and aghast, watching the disaster helplessly. “What’s going to happen to us? What will become of us?” During this pandemonium, only one tiny hummingbird was busy, going to get a few drops of water in its beak to throw on the fire. He flew back and forth non-stop from the river to the blaze. After a while, the armadillo, irritated by the hummingbird’s pathetic efforts, said: “Are you a fool! You don’t believe that with these drops of water you’re going to put out the fire!“The hummingbird responded:”I know I won’t, but I’m doing my share”* [44].

## Abbreviations

ILAE: International league against epilepsy
NGS: Next-Generation sequencing
WES: Whole-Exome sequencing
WGS: Whole-Genome sequencing
WHO: World Health Organization
